# Diversities and Shifts of Microbial Communities Associated with Farmed Oysters (*Crassostrea gigas*) and Their Surrounding Environments in Laoshan Bay Marine Ranching, China

**DOI:** 10.3390/microorganisms11051167

**Published:** 2023-04-29

**Authors:** Guangjie Fang, Haolin Yu, Yazhou Zhang, Jun Liang, Yanli Tang, Zhenlin Liang

**Affiliations:** 1 Zhejiang Marine Fisheries Research Institute, Zhoushan 316021, China; 2 Key Laboratory of Sustainable Utilization of Technology Research for Fishery Resources of Zhejiang Province, Zhoushan 316021, China; 3 Scientific Observing and Experimental Station of Fishery Resources for Key Fishing Grounds, Ministry of Agriculture and Rural Affairs of the People’s Republic of China, Zhoushan 316021, China; 4 Institute of Oceanology, Chinese Academy of Sciences, Qingdao 266071, China; 5 Fisheries College, Ocean University of China, Qingdao 266003, China; 6 Marine College, Shandong University, Weihai 264209, China

**Keywords:** pacific oyster, bacteria, protists, different growth phases, mass death

## Abstract

Pacific oysters (*Crassostrea gigas*) are widely cultured in Chinese marine ranching with high economic value. However, mass death of farmed oysters has occurred frequently in recent years because of diseases and environmental disturbance (e.g., high temperatures). In order to analyze the potential relationships between microorganisms and the death of farmed oysters, we compared the dynamics of bacterial and protist communities in oysters at different growth phases using high-throughput sequencing. The results showed that the microbial communities in farmed oysters significantly changed and were markedly different from microbes in natural oysters and the surrounding environments. The number of biomarker taxa among farmed oysters and their surrounding environments decreased gradually with the growth of oysters. During the mass death of farmed oysters, the microbial communities’ abundance of ecological function genes changed, and the correlations among microorganisms disappeared. These results enrich our understanding of the dynamics of microbial communities in farmed oysters at different growth phases, illustrating the characteristics of interactions among microorganisms during the mass death of farmed oysters. Our study is beneficial to promote the healthy aquaculture of oysters.

## 1. Introduction

Oysters (phylum Mollusca, family Ostreidae) are sessile benthic metazoans widely distributed in global intertidal zones. Oysters are filter-feeding bivalves with important ecological functions including water purification and habitat restoration, especially in oyster reefs around the coast [1]. As one of the world’s most widely cultured marine bivalves with high economic value, oysters have become an important source of protein for humans [2]. The Pacific oyster (*Crassostrea gigas*) is the most productive economic shellfish in the world because of its characteristics of strong adaptability, high fecundity, fast growth, and rich nutrition, and its production in China ranks first in the world [3,4]. In view of the prominent role of oysters, the influences of environmental factors on oyster genetics, breeding, aquaculture, and diseases have been the focus of numerous studies [5,6,7].

Microbial communities play crucial roles in the growth and development of macro-organisms, and are closely linked with oyster aquaculture [8,9,10]. Bacterial communities associated with different oyster tissues have previously been studied, in research which has mainly focused on microorganisms in the stomach [11,12], gonads [8], gills [13], and total organisms [14,15]. Some studies have addressed the different growth phases of oysters. Microbes are indispensable for the growth and health of oysters at different growth phases, and understanding the changing patterns of microbial communities is valuable for the aquaculture of oysters [16,17]. Many studies have concentrated on the dynamics of bacterial communities in the surrounding environments in response to oyster-farming activities [9,18]; some reports have compared bacterial communities between oysters and their adjoining habitats [19,20].

Previous studies stated that oysters’ microbiomes and their surrounding environments (seawater and sediment) are closely related, and the interactions among these biotopes are critical [21,22,23]. On the one hand, the suspension and sedimentation of sediment in mariculture areas pronouncedly affected oysters’ microbial communities [24]. On the other hand, with the intensification of aquaculture activities, the diversity and structure of the bacterial community in the closing sediment tended to change significantly [25]. Some studies also reported that sediment suspension is a vital interaction process for microbial communities between the water and sediment [26]. However, studies simultaneously profiling the bacterial and protist communities associated with farmed oysters and their surrounding environments are lacking.

Ecological symbiosis and interactions among bacteria and protists exist in all marine ecosystems and individual organisms [27,28,29]. These two types of microorganisms are crucial for nutrient cycling, transformation, and degradation [30]. Earlier studies also reported that organism-associated microbes contribute to the growth and health of their host macro-organisms [31,32]. Regarding bacteria, many predicted functions are significantly correlated with bacterial communities, including varied metabolisms, disease resistance, and immune responses [33]. Protists are key components in microbial loops as primary producers, consumers of bacteria, decomposers, and parasites [34], and are highly relevant to the health of halobios [35]. Despite the important ecological roles of bacterial and protist communities in the cultured species, changes in the relationships among bacteria, protists, and macro-organisms have frequently resulted in various diseases, and even the death of the hosts [36,37,38].

Laoshan Bay marine ranching is located in the Yellow Sea, China, where the mariculture of *C. gigas* using natural juveniles is practiced annually. Massive reproduction of oysters is observed from June to August, and sufficient free-swimming larvae are available to develop as sessile juveniles for aquaculture in Laoshan Bay (Figure S1). The oyster-farming procedures of local fishermen are as follows: (1) twenty scallop shells are tied to a 2 m nylon cord, and 30 cords are arranged as a group; (2) groups with an interval of 1.5 m are bound to the main rope, which is fixed on the surface of the seawater; (3) the main rope is kept 2 m below the water’ surface. It is recognized that the oyster aquaculture industry has for a long time been facing infectious diseases caused by microorganisms, which have dramatically affected the growth and yield of the oysters [39]. Due to the rapid spread of marine pathogens on a large scale, and limited ability to isolate oysters from invasion by pathogens in seawater, mass mortality of farmed oysters often occurs. Meanwhile, the natural oysters in the adjacent sea areas are healthy. These inconsistencies have always puzzled researchers. With the rapid advancement of high-throughput sequencing technologies, the profiling of microbiotas in the oysters has become more comprehensive [16]. However, our understanding of the internal mechanisms resulting in the mass death of farmed oysters is still limited.

In this study, we attempted to interpret the microbiome’s dynamics to better understand the attachment, growth, and death of the farmed oysters. The main objectives of this study were (1) to characterize the bacterial and protist communities of farmed oysters in three growth phases; (2) to compare the microbial communities between the farmed oysters and their surrounding environments (seawater and sediment); (3) to analyze the differences of microbial community compositions in diseased oysters and healthy oysters, and to explore the reasons for massive death of farmed oysters.

## 2. Material and Methods

### 2.1. Study Areas and Sample Collection

Raft culture of oysters was carried out on 6 July 2020, using scallop shell (*Argopecten irradians*) as artificial substrate. Fresh samples of oysters along with surrounding water and sediment samples were collected on 21 July (15 days, oyster larvae were visible on the scallop shells) and 22 August (48 days, mean shell length of oysters was 38.7 mm). As a control, wild populations of oysters in the intertidal zone and on the seafloor were sampled on 22 August. Massive death of farming oysters occurred in October, and the diseased samples were obtained on 12 October. All samples were immediately frozen on ice and transported back to the laboratory within 2 h. Microbial biofilms on the surfaces of oysters were thoroughly rubbed with sterile brushes and rinsed with sterile seawater. The rinsing samples and water samples were filtered through a 0.2 µM pore 47 mm polycarbonate filter (PF, Millipore), and were then stored at −80 °C for DNA extraction. Sediment samples were stored at −80 °C for later analysis.

### 2.2. DNA Extraction, PCR Amplification, and Illumina MiSeq Sequencing

Genomic DNA was extracted from the triplicate samples using the FastDNA^®^ SPIN kit for soil (MP Biomedicals, Irvine, CA, USA) according to the manufacturer’s instructions. The DNA extract was checked on a 1% agarose gel, and the DNA concentration and purity were determined using a NanoDrop 2000 UV-vis spectrophotometer (Thermo Scientific, Wilmington, DE, USA). The extracted DNA samples were PCR amplified using two sets of primers. One was 338F (5′-ACTCCTACGGGAGGCAGCAG-3′) and 806R (5′-GGACTACHVGGGTWTCTAAT-3′), which amplified the V3-V4 hypervariable region of the 16S rRNA for bacteria, and the other was V4F (5′-CCA GCA SCY GCG GTA ATT CC-3′) and V4RB (5′-ACT TTC GTT CTT GAT YRR-3′) [40], which amplified the V4 hypervariable region of the 18S rRNA for protists. The PCR amplification procedures complied with the standard guidelines of the sequencing protocol.

### 2.3. Sequence Analysis

Raw sequence reads were analyzed using the QIIME 1.9 bioinformatics pipeline. Sequences shorter than 200 bp, those with quality scores lower than 20, and those with mismatched bases in barcodes or primers were removed. The operational taxonomic units (OTUs) were clustered with a 97% similarity cutoff using UPARSE 7.0, and chimeric sequences were identified and removed using UCHIME. The taxonomy of each sequence was analyzed by RDP Classifier 11.5 against the Silva database (v138). For downstream analyses, a randomly selected subset of the lowest sequencing number per sample was used to compare the relative differences between samples. Sequencing data are publicly available in the National Center for Biotechnology Information (NCBI) Sequence Read Archive (SRA) database under accession of PRJNA797707 and PRJNA797713.

### 2.4. Statistical Analysis

Alpha diversity estimators (OTU, Shannon, Chao 1) were calculated and compared among all samples. Venn diagrams were applied to analyze the numbers of OTUs in samples (package *VennDiagram*). Principal coordinate analysis (PCoA) was performed to compare the microbial community compositions according to the relative abundance data based on a Bray–Curtis distance matrix (package *phyloseq*), and the significant difference test was carried out by analysis of similarity (ANOSIM) (package *vegan*). Ternary phase diagrams were used to show the relative abundance of microbial species in different samples. Biomarker taxa significantly associated with specific samples were identified using linear discriminant analysis (LDA) and were considered significant with an LDA score of at least 4.0 [41]. The functional annotations of bacteria and protists were implemented using the PICRUSt2 package with the MataCyc pathway database. Statistical differences were analyzed using the Kruskal–Wallis test, and were considered significant at *p* < 0.05. Statistical analyses were conducted in R 4.0.2.

## 3. Results

### 3.1. Variations of the Microbial Community Diversities in Oysters

**Figure 1:**
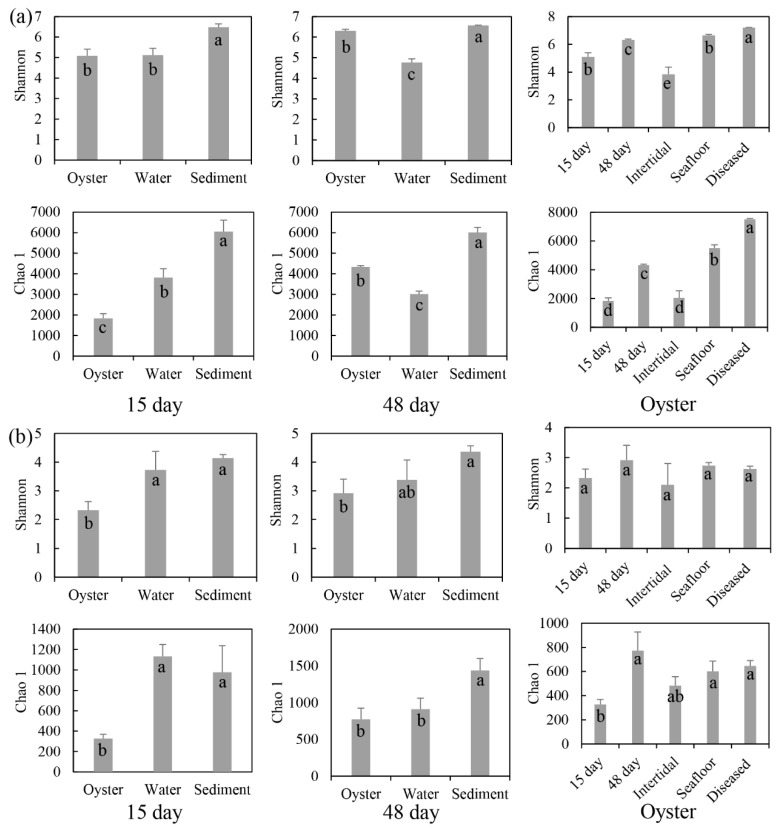
Diversity indices (mean ± SD) of (**a**) bacterial and (**b**) protist communities associated with oysters. 15 day: farmed oysters and their surrounding environmental samples obtained after 15 days of aquaculture. 48 day: samples obtained after 48 days of aquaculture. Oyster: farmed oysters after 15 days and 48 days, and diseased; and natural oysters in the intertidal zone and on the seafloor. Lower-case letters in the bars indicate significant differences analyzed by the statistical test.

Microbial community compositions of the oyster biofilms, water, and sediment were compared. After 15 days of cultivation, the number of OTUs ranged from 2223 (oysters) to 6430 (sediment) for the bacterial samples, and from 497 (oysters) to 1633 (sediment) for the protist samples (Figure S2). After 48 days of cultivation, the number of bacterial and protist OTUs in oysters were 4351 and 941, respectively. The microbial communities’ Alpha diversity indices (Shannon and Chao 1) of microbial communities are illustrated in Figure 1. After 15 days of cultivation, the bacterial Shannon diversity of sediment was significantly higher than that of oysters or water; the bacterial Chao 1 diversity of oysters was lowest, and that of sediment was highest. However, for protists, the Shannon and Chao 1 diversity indices of oysters were both lower than those of water or sediment. After 48 days of cultivation, the bacterial Shannon and Chao 1 diversity indices of oysters were higher than those of water, and lower than those of sediment. For protists, the Shannon and Chao 1 diversity indices of oysters and water were significantly lower than those of sediment.

Microbial biofilms of farmed oysters and two natural oysters from the intertidal zone and seafloor were analyzed, and then compared with the α diversity of farmed oysters in the three growth phases. For the farmed and natural oysters, the number of bacterial OTUs ranged from 2144 (intertidal zone) to 5569 (seafloor), and the number of protist OTUs in the farmed oysters was higher than in two natural oysters (Figure S2). The Shannon and Chao 1 diversity indices of oysters in the intertidal zone were lowest for bacterial and protist communities. Those of oysters on the seafloor were highest for bacteria and those of farmed oysters were highest for protists. For the three growth phases, the OTUs of bacterial communities varied from 2223 (15 days) to 7450 (diseased), and protist OTUs changed from 497 (15 days) to 941 (48 days). The Shannon and Chao 1 diversity indices for bacteria increased with aquaculture days. However, for protists, two indices increased from 15 to 48 days, then decreased in diseased oysters (Figure 1).

### 3.2. The Oyster-Associated Microbial Community Compositions

**Figure 2:**
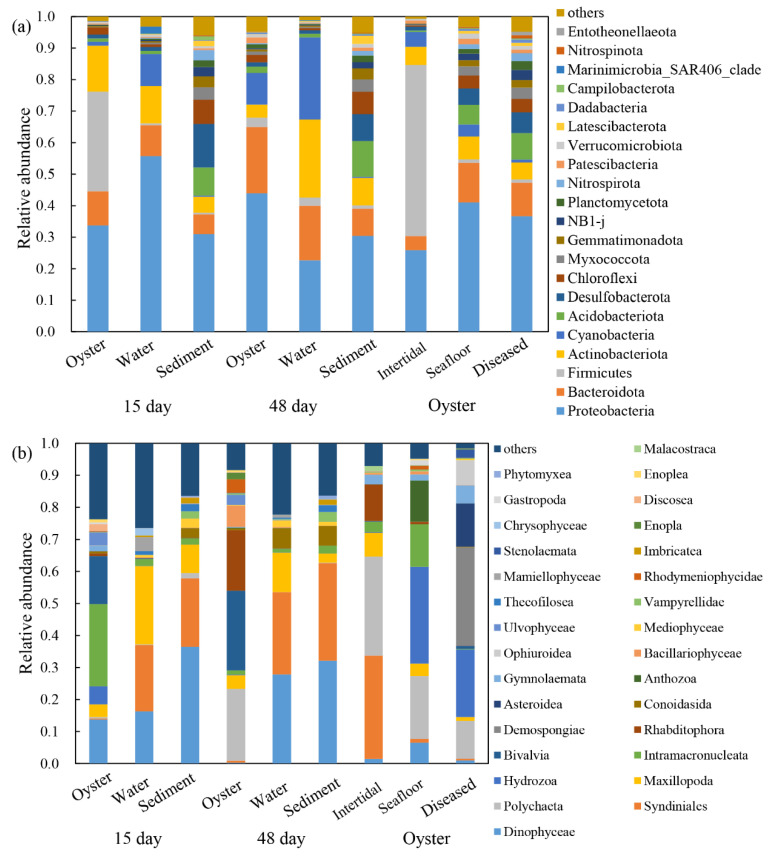
Relative abundance of (**a**) bacterial communities at phylum level and (**b**) protist communities at class level in the oysters and their surrounding environments. 15 day: farming oysters, water, and sediment samples obtained after 15 days of aquaculture. 48 day: samples obtained after 48 days of aquaculture. Oyster: farmed oysters when diseased, natural oysters in the intertidal zone and seafloor.

After 15 days of cultivation, the bacterial communities of oyster biofilms, water, and sediment were dominated by the phyla Proteobacteria (33.7%), Firmicutes (31.7%), Actinobacteriota (14.6%), and Bacteroidota (10.8%); phyla Proteobacteria (55.7%), Actinobacteriota (11.9%), and Cyanobacteria (10.2%); phyla Proteobacteria (31.0%) and Desulfobacterota (13.8%), respectively (Figure 2a). The dominant protist communities were class Intramacronucleata (25.7%), Bivalvia (15.0%), and Dinophyceae (13.7%); class Maxillopoda (24.5%), Syndiniales (20.7%), and Dinophyceae (16.3%); class Dinophyceae (36.5%) and Syndiniales (21.4%), respectively (Figure 2b). After 48 days of cultivation, the top three dominant bacterial phyla for three samples were Proteobacteria (44.0%), Cyanobacteria (26.1%), and Proteobacteria (30.4%), and the dominant protist classes were Bivalvia (24.9%), Dinophyceae (27.9%), and Dinophyceae (32.1%).

The most dominant bacterial phylum in farmed oysters (48 days) was Proteobacteria (44.0%), while Firmicutes (54.3%) and Proteobacteria (41.1%) were predominant in the bacterial communities of the two wild oysters (Figure 2a). The most dominant protists of the three types of oysters were class Bivalvia (24.9%), Syndiniales (32.3%), and Hydrozoa (30.2%), respectively (Figure 2b). As for the dominant species in farmed oysters in the three growth periods, the bacterial communities were consistent (phylum Proteobacteria) with relative abundance of 33.7%, 44.0%, and 36.6%; while the most dominant protist classes changed from Intramacronucleata (25.7%) and Bivalvia (24.9%) to Demospongiae (30.8%).

**Figure 3:**
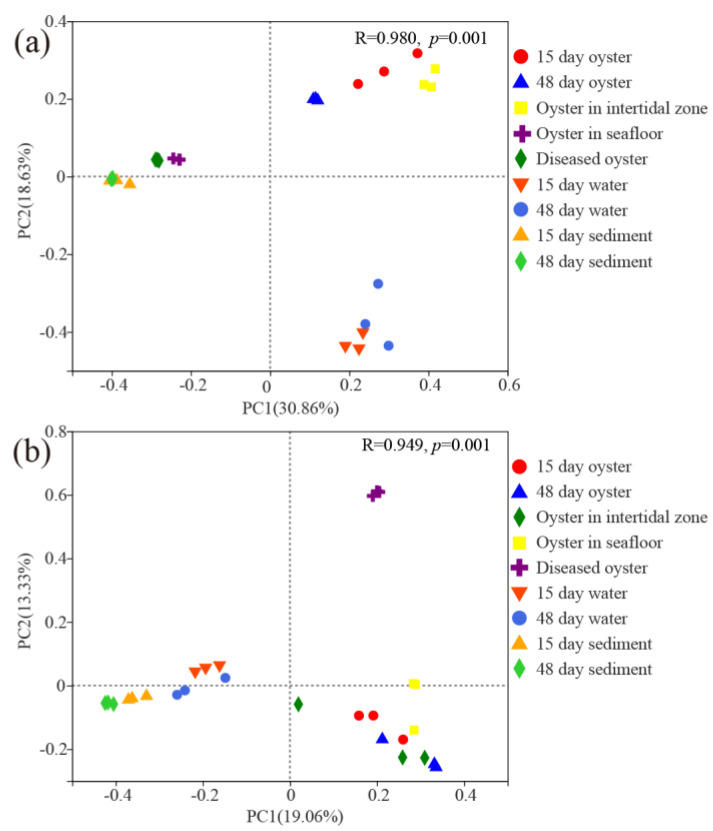
Principal coordinate analysis (PCoA) plots of (**a**) bacterial communities and (**b**) protist communities associated with farmed oysters and surrounding environments.

Three distinct groups were observed based on the results of PCoA both for the bacterial and protist communities (Figure 3). For bacterial communities (ANOSIM, *p* < 0.001), the first and second principal coordinates explained 30.86% and 18.63% of the variations in the community compositions. The three groups were as follows: the first two growth phases of farmed oysters and oysters in the intertidal zone were group I; diseased oysters, oysters on the seafloor, and sediment were group II; water samples were group III. For protist communities (ANOSIM, *p* < 0.001), the first and second principal coordinates explained 19.06% and 13.33% of the variations in the community compositions. The diseased oysters comprised group I; water and sediment samples were classified as group II; other oyster samples were regarded as group III.

### 3.3. Biomarker Taxa and Potential Functional Profiling of Oyster-Associated Microbial Communities

After 15 days of cultivation, biomarker taxa in the farming oysters were observed, including bacterial classes Bacilli and Actinobacteria, genera *Erythrobacter* and *Planococcus*, and protist classes Intramacronucleata, Bivalvia, and Hydrozoa, genera *Karlodinium* and *Acineta* (Figures S3 and S4). After 48 days of cultivation, bacterial class Alphaproteobacteria, genera *Erythrobacter* and *Lewinella* were the biomarker taxa in the oysters; protist classes Bacilllariophyceae, Bivalvia, Polychaeta, and Rhabditophora, genera *Navicula* were the most frequent specific species. Regarding the three periods of farmed oysters, particular biomarker taxa were found in the diseased oysters. For example, bacterial genera *Woeseia* was the only specific species observed in the diseased oysters; protist biomarker taxa were more diversified, including classes Gymnolaemata, Hydrozoa, Ophiuroidea, and Demospongiae, family Phyllopharyngea.

**Figure 4:**
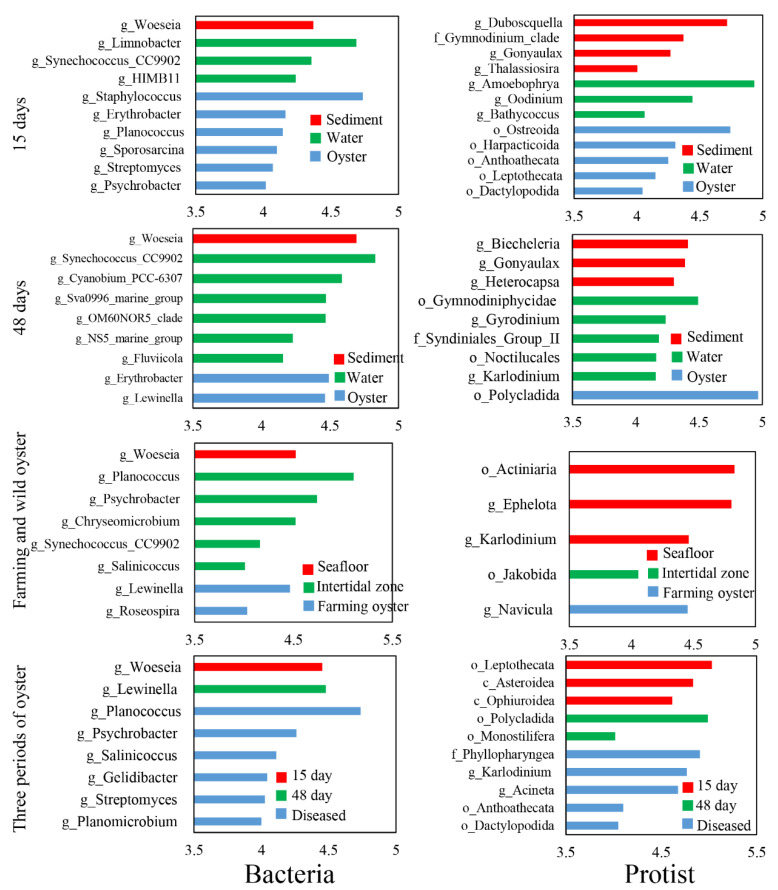
Biomarker taxa at genus level with a linear discriminant analysis (LDA) score of more than 4.0.

Results of LDA showed six and two genera with LDA scores more than 4.0 for bacteria in the farming oysters after 15 and 48 days of cultivation, respectively, which were significantly different from the water and sediment samples (Figure 4). For protists, five and one genera in the farming oysters were identified as biomarker taxa after 15 and 48 days of cultivation (Figure 4), resecptively. The abundance of two bacterial genera (*Lewinella* and *Roseospira*) and one protist genus (*Navicula*) significantly differed from those in the two natural oysters. In contrast to the other two farming phases, one and three biomarker taxa at genus level for bacteria and protists, respectively, were discovered in diseased oysters.

**Figure 5:**
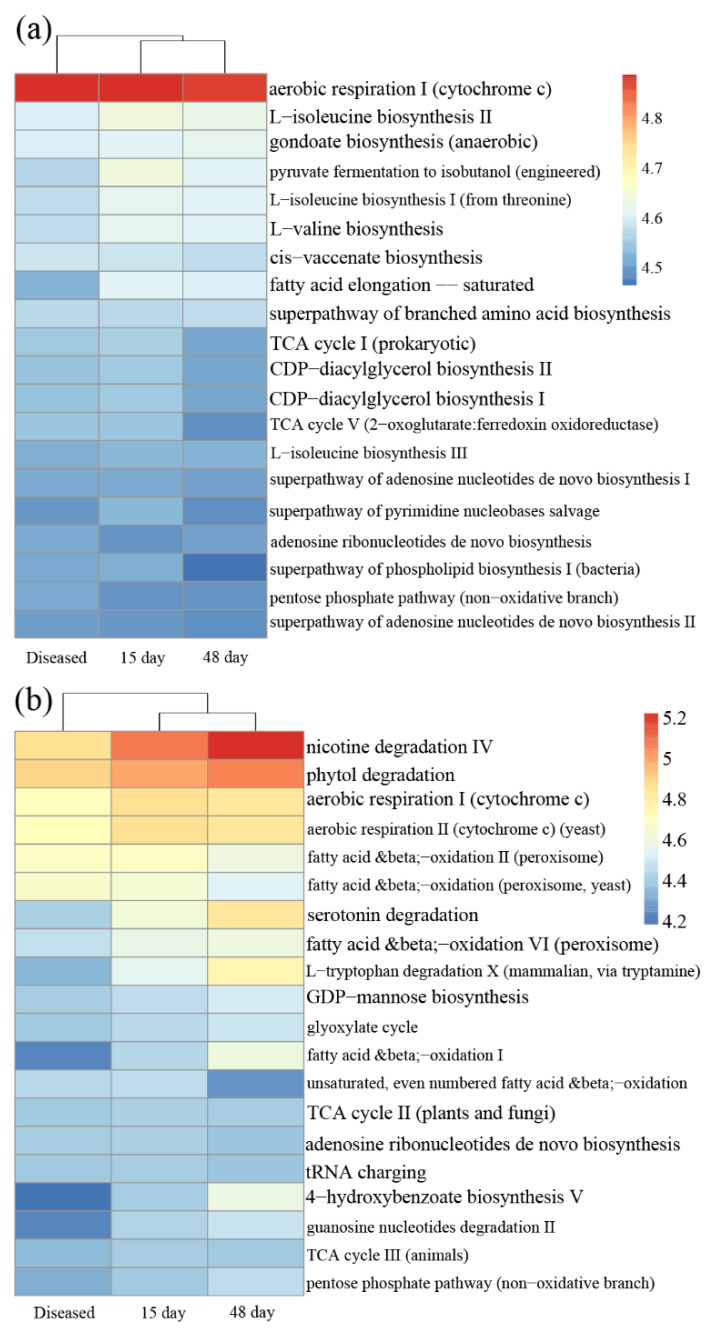
Heatmap of predicted functions investigated in (**a**) bacterial communities and (**b**) protist communities, based on the MetaCyc pathway database. Diseased: microbial communities in the diseased farmed oysters; 15 day: after 15 days in farmed oysters; 48 day: after 48 days in farmed oysters.

The PICRUSt2 analysis indicated that potential functional annotations of three phases of farming oysters were divided into two groups both for bacterial and protist communities: oysters after 15 days and 48 days of cultivation as group I; diseased oysters as group II (Figure 5). For bacteria, functions relating to synthesis amino acids (e.g., L-isoleucine biosynthesis and gondoate biosynthesis) were much lower in diseased oysters than in the other two healthy oysters. For protists, the abundance of three functions (nicotine degradation IV, phytol degradation, and aerobic respiration) was lowest in the diseased phase.

### 3.4. The Correlations of Microbial Communities

The correlations between bacteria and protists associated with oysters were analyzed (Table 1). After 15 days of cultivation, 2 bacterial OTUs and 29 protist OTUs were observed with 188 significant correlations, and 93% occurred among protist OTUs. After 48 days of cultivation, 3 bacterial OTUs and 9 protist OTUs were observed in the farmed oysters, and 81% of the total 37 correlations were found among protist OTUs. For the natural oysters in the intertidal zone and on the seafloor, 13 microbial OTUs were detected in both, and the proportions of correlations among bacteria and protists were consistent. Totals of 6 and 16 microbial OTUs were discovered in the water and sediment samples, and nearly 70% correlations existed among protist OTUs. However, no significant relationship was observed in the diseased oysters. the highest proportion of positive correlations among microorganisms was observed in the farmed oysters after 15 days (100%), followed by the farmed oysters after 48 days, natural oysters in the intertidal zone, sediment samples (all over 90%), water samples (75%) The proportion in natural oysters on the seafloor was lowest (66%).

## 4. Discussion

### 4.1. Microbial Communities in the Farming Oysters at Different Growth Phases

The Pacific oyster (*Crassostrea gigas*) is one of the most important aquaculture species in China, and the raft culture of oysters using scallop shells as artificial substrate is widely practiced by fishermen. Biofilms on the shells are vital for the attachment and growth of *C. gigas*, and some infectious diseases originate here. In the present study, bacterial and protist communities in the biofilms of farming oysters at different growth phases, diseased oysters, natural oysters, and their surrounding environments were compared using high-throughput sequencing.

After 15 days of cultivation, larvae of *C. gigas* had attached to the shells; after 48 days, *C. gigas* had greatly increased in size. The alpha diversity indices of bacterial and protist communities associated with farming oysters at two growth phases (15 and 48 days) were inconsistent (Figure 1 and Figure S2). Shannon and Chao 1 indices observed after 15 days were significantly lower than after 48 days. After the deployment of raft cultures, *C. gigas* began to attach to the shells, which started changing the microbial diversities of biofilms on shells. With the growth of *C. gigas*, diversities of microbial communities varied, increased, and ultimately stabilized. Bacteria phyla Firmicutes and Actinobacteriota were more abundant after 15 days, and phyla Bacteroidota and Cyanobacteria were more enriched after 48 days (Figure 2). On the basis of the evidence, we deduced that the function of bacterial communities (15 days) is more focused in the attachment phase, and then gradually changes during growth (48 days). Phyla Firmicutes and Actinobacteriota were consistently detected in the sediment, indicating that the early stage of oyster attachment might have a high correlation with sediment microorganisms [22]. We also supposed that suspended particulate matter from sediment provides essential nutrition for the larval *C. gigas*. The protist classes Dinophyceae and Intramacronucleata showed higher abundance after 15 days, while classes Polychaeta and Rhabditophora were richer after 48 days. Two reasons may explain the dynamics of protist communities. First, oysters are filter feeders, their feeding preferences change with their growth phases [42,43]. Second, with the enlargement in size of individual oysters, their shells become ideal habitats for marine organisms, and the dominant species of protists change from eukaryotic microalgae to protozoa.

### 4.2. Microbial Communities of the Farming Oysters and Natural Oysters

Variations of habitats could result in considerable differences in microbial communities [23]. The Shannon diversity of bacterial communities of natural oysters on the seafloor was higher than that of farmed oysters, and that of natural oysters in the intertidal zone was lowest (Figure 1a). As for the protist communities, there were no significant differences among oyster samples (Figure 1b). These results revealed that environmental disturbance such as intense light and tidal variation had a greater influence on the microbial communities of natural oysters in the intertidal zone. The differences in Shannon diversity of bacterial communities among oyster samples were higher than the diversity of protist communities after 48 days of cultivation, showing that bacterial communities in the oysters were more sensitive to habitat variations than were the protist communities.

Microbial diversity assessment is considered one of the critical elements in analyzing the feasibility of marine aquaculture systems [6]. An in-depth study of the microbial community differences between farmed and natural oysters is conducive to monitoring and evaluating whether the microbial community has reached a balance, which is vital to maintain the health of oysters and inhibit colonization by oyster pathogens [44]. Our results showed that the dominant species and microbial compositions were inconsistent among farmed and natural oysters. The filter-feeding habits and varied habitats of the oysters may have caused these differences. Previous studies have revealed that oysters have a strong filtering ability; about of 75% microorganisms remain in the oysters during the digestive process and form a dynamic balance of new communities and original microbial communities [45]. Complex hydrological conditions and different environmental disturbances may also affect oysters’ microbial communities.

The edible quality of farmed oysters is highly correlated with the surrounding environment [46]. The results indicated that the α diversity of oyster microorganisms gradually approached or even exceeded the microbial diversity of water over time, but was lower than that of sediment (Figure 1). It could be inferred that the relationships of microbial communities between water and oysters are comparatively higher. Forrest et al. summarized the interactions between farmed oysters and the water environment and suggested that the lack of microbial monitoring of edible marine bivalves might pose a severe risk to human health [47]. Ortega et al. reported that oysters cultured in lower salinity have a higher degree of microbial pollution [48]. Souza et al. evaluated the microbial communities of farmed oysters in three water-quality environments and confirmed that the harmful pathogenic bacteria in oysters mainly come from marine water [45]. Above all, strengthening the microbial community monitoring of oysters and their surrounding environment can help ensure the edible quality of oyster products.

### 4.3. Microbial Communities in the Diseased Oysters

Many inducing factors for the massive death of oysters have been reported, among which infectious pathogens (such as bacteria, eukaryotic microorganisms, viruses, etc.) and environmental changes (such as high temperature, pollution, etc.) are the most important and common factors [49]. In recent decades, the phenomenon of massive death of oysters in summer due to high temperatures and for other reasons has occurred widely around the world, leading to high mortality and substantial financial losses [50]. Many studies have reported that high temperature may induce the immune response of oysters, promoting oysters to release a quantity of energy and therefore leading to the decline of oysters’ immunity and even their death [51]. However, the seawater temperature in Laoshan Bay has been consistent in recent years, and no evident rise in temperature was observed in this study. Considering the experiences of fishermen, the massive death of oysters may be related to microbial pathogens.

Common pathogens that cause oysters’ death include bacteria (e.g., *Vibrio*), protists (e.g., *Sirolpidium*), and viruses [52,53]. The results of the co-occurrence network analysis revealed that protists played dominant roles in the microbial communities of diseased oysters (Table 1), implying a stronger correlation between the protists and the massive death of oysters. Furthermore, more protist biomarker taxa were observed compared with bacteria (Figure 4 and Figure S3). For instance, *Stegotricha enterikos* belongs to the class Phyllopharyngea, phylum Ciliophora, and caused the disease of Pacific oysters in British waters [54]. However, our understanding of marine protists lags far behind our knowledge of bacteria, and many remain to be discovered [55]. The PICRUSt2 results showed that some crucial functional gene abundances were significantly lower in diseased oysters than healthy oysters (Figure 5). It can be seen that the ecological service provided by the microbes in oysters changed substantially during the period of disease. Our study confirmed a potential correlation between the health of oysters and the interactions of microorganisms. Disordered interactions of microbial communities could lead to the loss of microbial community functions and even the death of the hosts. Shellfish aquaculture activities in China are mainly carried out in shallow waters, intertidal zones, and other open sea waters. It is impossible to change or even control the environmental microbial communities in mariculture areas through technical methods, such as introducing drugs or improving water quality. Hence, monitoring the microbial community compositions of oysters, surrounding seawater, and sediments, and regularly estimating the abundance of common pathogenic microorganisms, are valuable for alert against oyster diseases. These monitoring processes are of guiding significance for the effective prevention and control of oyster diseases.

## 5. Conclusions

In the present study, we compared the composition of bacterial and protist communities in oysters at different growth phases, in different habitats, in healthy and diseased oysters, and in their surrounding environments. With the growth of oysters, the α diversity of microbial communities in the oysters had high similarity with that in surrounding seawater. However, the microbial community compositions could be divided into three groups. Accordingly, bacterial and protist communities in oysters were found to have different mechanisms of community dynamics at different periods. The analysis results of microbial biomarker taxa and potential community function indicated that the microbial communities of diseased oysters differed significantly from those of healthy oysters. Furthermore, the co-occurrence networks confirmed that the interactions among the microbial communities of diseased oysters reduced significantly, and the correlation between protist communities and disease in oysters was higher than that for bacterial communities. We suspect that changes in the interactions among microorganisms are related to oyster death, especially with regard to protists. In order to reduce the losses caused by diseases in the oyster-farming industry, it is recommended to regularly monitor microbial communities and establish an early warning mechanism. Our study preliminary analyzed the microbial community dynamics of the oysters and their surrounding environment, and further studies are suggested to provide a more systematic comparative study of different tissues of oysters. Moreover, applying advanced molecular biological methods to improve protist-identification ability is encouraged.

## Figures and Tables

**Table 1:** The correlations of bacteria and protists associated with oysters. Numbers and proportions of OTUs and correlations were analyzed. B-P: correlations between bacteria and protists; B-B: correlations between bacteria and bacteria; P-P: correlations between protists and protists.

Sample	OTUs			Correlations			
	Bacteria	Protist	Total	B-P	B-B	P-P	Total
Farmed oysters after 15 days	2	29	31	10	3	175	188
	0.06	0.94	1.00	0.05	0.02	0.93	1.00
Farmed oysters after 48 days	3	9	12	3	4	30	37
	0.25	0.75	1.00	0.08	0.11	0.81	1.00
Natural oysters in intertidal zone	4	9	13	14	7	19	40
	0.31	0.69	1.00	0.35	0.18	0.48	1.00
Natural oysters on the seafloor	5	8	13	15	9	23	47
	0.38	0.62	1.00	0.32	0.19	0.49	1.00
Diseased oysters	-	-	-	-	-	-	-
Water	1	5	6	4	1	11	16
	0.17	0.83	1.00	0.25	0.06	0.69	1.00
Sediment	4	12	16	15	5	51	71
	0.25	0.75	1.00	0.21	0.07	0.72	1.00

## Data Availability

MDPI Research Data Policies.

## Supplementary Materials

The following supporting information can be downloaded at: https://www.mdpi.com/article/10.3390/microorganisms11051167/s1, Figure S1: Map of sampling areas in the Laoshan Bay, China; Figure S2: Venn diagrams revealing the numbers of shared and unique OTUs for (a) bacterial communities and (b) protist communities; Figure S3: Ternary phase diagrams showing biomarker taxa at class level and genus level for bacterial communities; Figure S4: Ternary phase diagrams showing biomarker taxa at class level and genus level for protist communities.
