# Supplementary material for: Diversities and Shifts of Microbial Communities Associated with Farmed Oysters (Crassostrea gigas) and Their Surrounding Environments in Laoshan Bay Marine Ranching, China
Source: Microorganisms. 2023 Apr 29;11(5):1167. doi: 10.3390/microorganisms11051167 (PMC10222713; doi:10.3390/microorganisms11051167)
Supplement: Supplementary file 1 [file microorganisms-11-01167-s001.zip › microorganisms-2293209-supplementary.pdf]

microorganisms

MDPI

Supplementary materials

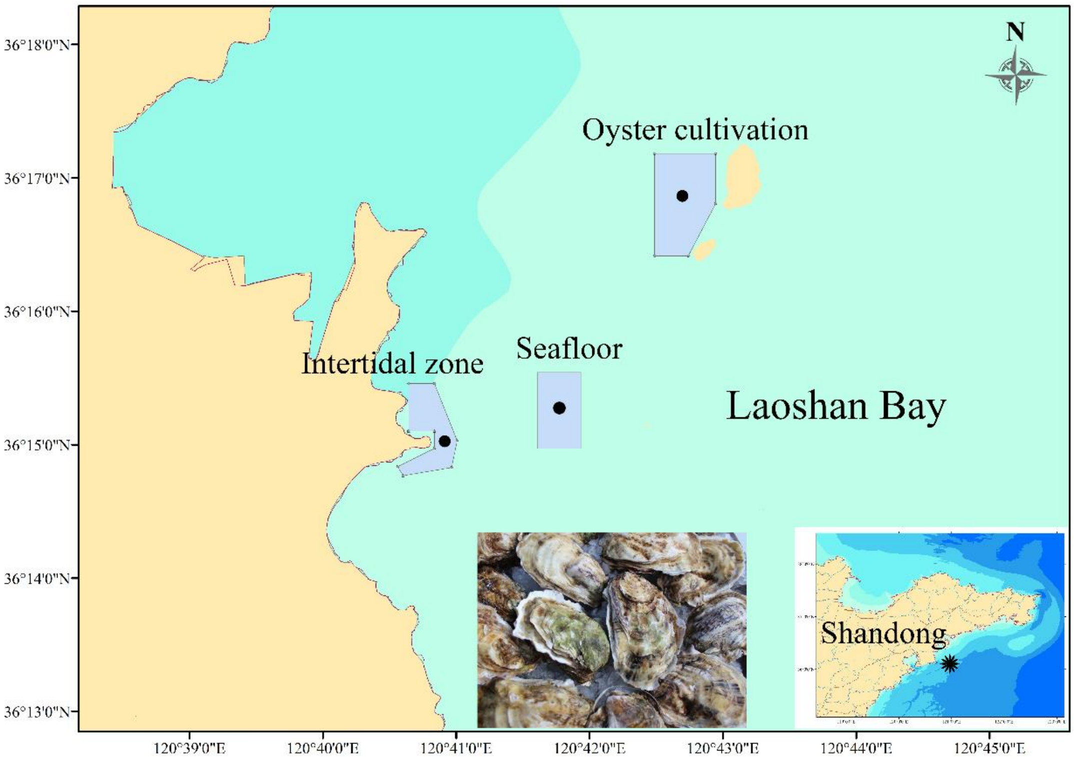

Figure S1. Map of sampling areas in the Laoshan Bay, China. Farming oysters were sampled from oyster cultivation zone, natural oysters were obtained from intertidal zone and seafloor zone. Laoshan Bay marine ranching is located in the Laoshan Bay, Yellow Sea, China, which has a high-quality aquatic environment and fishery resources. The mouth of Laoshan Bay faces south and is open. The bay covers an area of 164 square kilomters, and the maximum water depth is about 13 m. The bay belongs to the regular semi diurnal tidal zone, with a maximum tidal range of about 4.2 meters. There is a large area of intertidal shoals at the top of the bay.

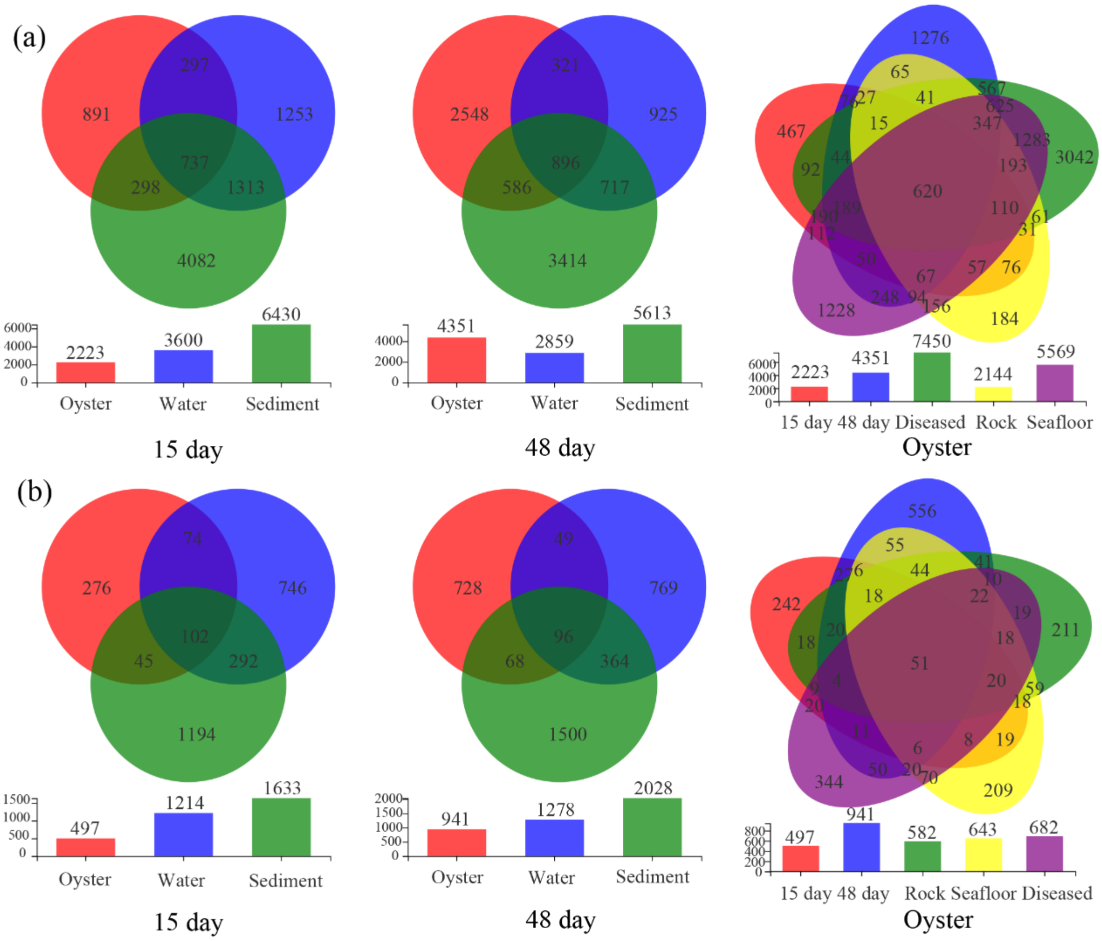

Figure S2. Venn diagrams revealing the numbers of shared and unique OTUs for **(a)** bacterial communities and **(b)** protist communities. 15 day: farming oysters and its surrounding environmental samples obtained after 15 days of aquaculture. 48 day: samples obtained after 48 days of aquaculture. Oyster: farming oysters after 15 days, 48 days and diseased, natural oysters in the intertidal zone and seafloor.

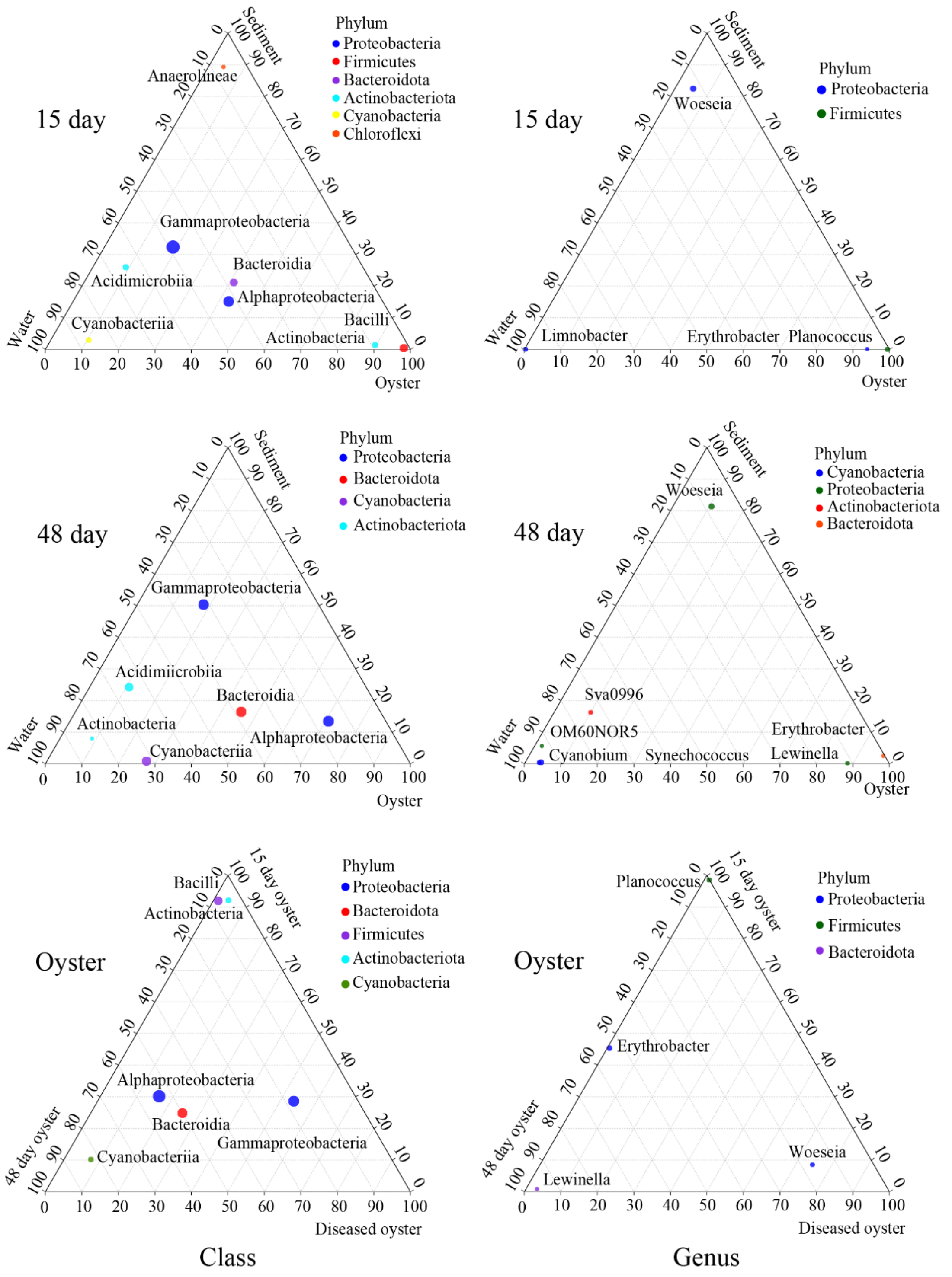

Figure S3. Ternary phase diagrams showing biomarker taxa at class level and genus level for bacterial communities. 15 day: farming oyster and its surrounding environmental samples obtained after 15 days of aquaculture. 48 day: samples obtained after 48 days of aquaculture. Oyster: farming oysters after 15 days, 48 days and diseased.

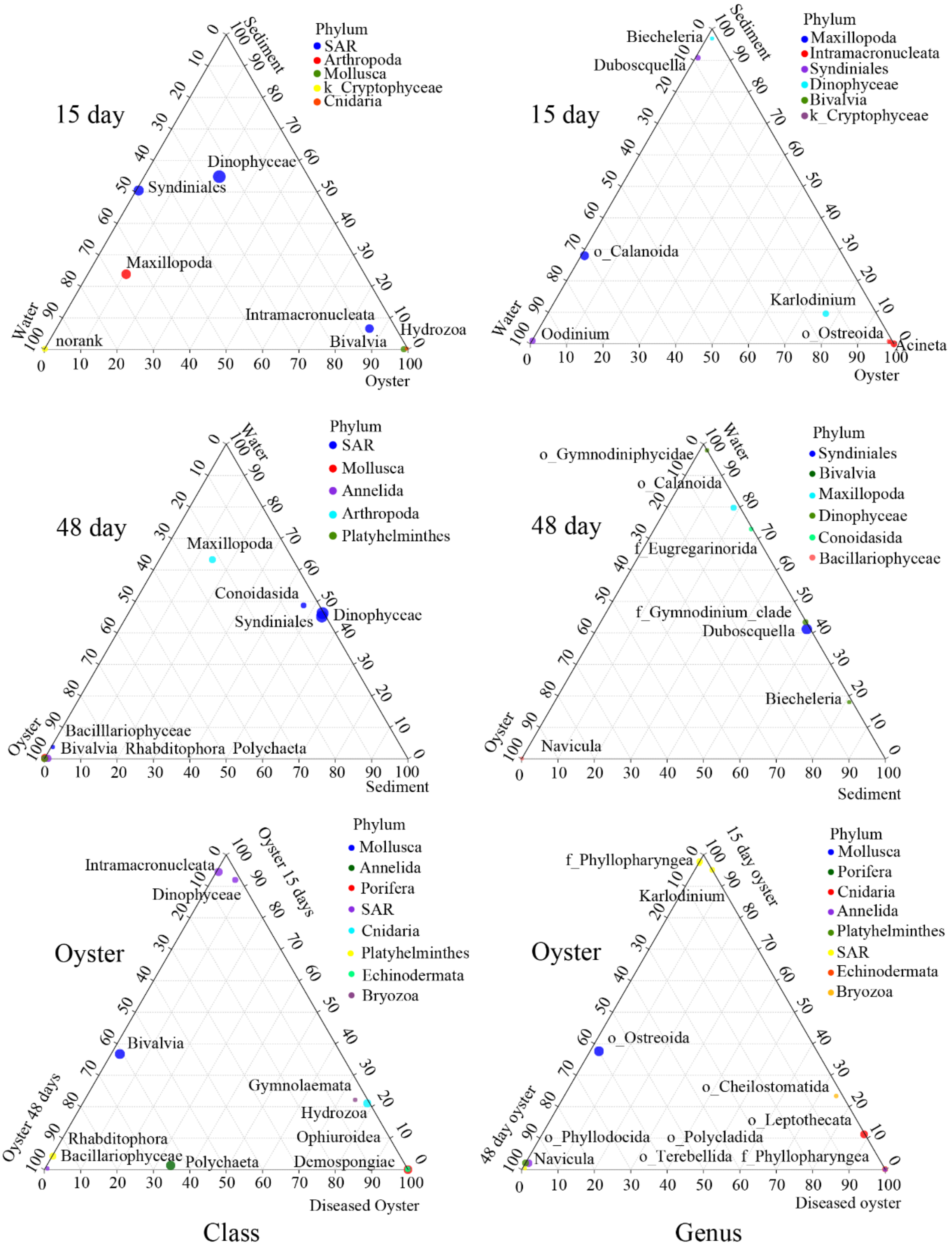

Figure S4. Ternary phase diagrams showing biomarker taxa at class level and genus level for protist communities. 15 day: farming oyster and its surrounding environmental samples obtained after 15 days of aquaculture. 48 day: samples obtained after 48 days of aquaculture. Oyster: farming oysters after 15 days, 48 days and diseased.
